# Total Fat and Fatty Acid Content in Meals Served by Independent Takeaway Outlets Participating in the Healthier Catering Commitment Initiative in London, UK

**DOI:** 10.3390/ijerph22010121

**Published:** 2025-01-18

**Authors:** Agnieszka Jaworowska, Susan Force

**Affiliations:** 1 Division of Medicine, Faculty of Medical Sciences, University College London (UCL), London WC1E 6BT, UK; 2 School of Science, Faculty of Engineering and Science, University of Greenwich, Chatham ME4 4TG, UK

**Keywords:** takeaway meals, out-of-home meals, fat content, saturated fatty acids, trans fatty acids, public health nutrition interventions

## Abstract

Out-of-home meals are characterized by poor nutritional quality, and their intake has been linked to adverse health outcomes. Therefore, national and local government initiatives have been implemented in the UK to promote healthier out-of-home meals. However, there is limited evidence of their effectiveness. This study evaluated the fat content and fatty acid profile of takeaway meals from ‘standard’ and from Healthier Catering Commitment (HCC)-approved takeaway outlets. Meals from 14 ‘standard’ and 13 ‘HCC-awarded’ takeaways (74 meals and 26 side dishes) were analyzed for total fat and fatty acid composition. No statistically significant differences in total fat, saturated, and trans fatty acids per 100 g and per portion between HCC and standard meals were observed, except for donner kebabs. Over 70% of all meals contained more than the recommended 30% of daily fat intake from a single meal. Some meals could provide more than 50% of the recommended total fat and SFAs intake. Despite businesses participating in the healthier out-of-home meal initiative, there has not been a significant improvement in the nutritional quality of the meals they offer. Further research to develop effective approaches to support independent takeaway businesses in offering meals with improved nutritional quality is warranted.

## 1. Introduction

Over the past several decades, dramatic changes in food consumption patterns have resulted in an increased intake of out-of-home meals [1]. Unfortunately, these unhealthy eating habits are overtaking healthy eating patterns all over the world, and out-of-home meals have become a regular and significant component of the Western diet [2]. According to UK NDNS 2008–2012 data, 27% of adults and 19% of children ate out in restaurants or cafes, while 21% of people consume takeaway meals at home, at least once a week [3]. More recent data from 2023 indicate that 80% of United Kingdom (UK) adults eat takeaway food at least once per month [4], and it is predicted that the proportion of individuals eating out regularly is going to increase further [5,6]. A similar frequency has been observed among US adults, with 33% reporting eating restaurant meals three times per week or more [7]. In Australia, takeaway food consumption at least once a week was reported for 31.7% and 18.0% of children and adults, respectively [8]. Commercially prepared meals provide around 28% and 34% of the total energy intake for Korean and US adults, respectively [9,10].

Out-of-home meals are characterized by a poor nutritional profile, including high energy, salt, total fat, saturated fatty acids, and sugar content [11,12,13,14,15,16]. For example, an analysis of 489 meals, including Indian, Chinese, English, Kebab, and Pizza, from small, independent takeaway outlets across North West England found that, in some cases, the consumption of only one meal would be enough to provide daily energy, most macronutrient requirements, and exceed the recommended intake for salt [11]. In addition, out-of-home eating habits have undesirable effects on overall diet quality, characterized by a higher consumption of highly processed meat, sugar sweetened beverages, and sweets and a lower intake of fruits, vegetables, whole grains, and dairy products [17,18,19]. This unfavorable consumption pattern has been identified as an important environmental factor contributing to negative health outcomes, such as an increased risk of hypertension, insulin resistance, type 2 diabetes, cardiovascular disease, and obesity [20,21,22,23]. In 2021, seven of the ten leading causes of death globally were noncommunicable diseases (NCDs) [24], and it is predicted that their burden will rise [25]. In addition, NCDs put significant pressure on the health care system and the overall economic system [26].

In recognition of the above, national and local government initiatives have been implemented in the UK to promote healthier out-of-home meals [27]. These interventions often use ‘health by stealth’ approaches such as reformulation, healthier cooking practices, and eliminating choices to improve the nutritional quality of takeaway meals [27]. In 2011, the UK government introduced the ‘Public Health Responsibility Deal’ (RD) food pledges to support food outlets in the provision of healthier out-of-home meals, although small independent food catering businesses have not participated as much as larger chain food outlets in the food industry [28,29]. The RD evaluation demonstrated that it did not meet its objectives to improve public health in England [30]. Therefore, in 2022, mandatory energy labeling was introduced in the UK for large food businesses (250 or more employees) that prepare and sell meals ready for immediate consumption [31]. Unfortunately, small independent takeaway/fast-food businesses are not subject to this regulation. Recent evidence indicates that consumers would like healthier food to be offered in a number of food settings, including takeaways (48% of consumers) and fast-food restaurants (46% of consumers). The majority of those surveyed were likely to purchase food reduced in sugar, fat, and salt but less likely to purchase food with reduced portion size [32].

With independent fast-food/takeaway operators accounting for 62% of total fast-food outlets in the UK [33], local authorities have introduced a range of multi-component voluntary award schemes. The emphasis of these schemes is on encouraging food businesses to provide healthier meals with minimal or no impact on cost and taste that makes them appealing and popular among consumers [34]. The Healthier Catering Commitment (HCC) for London is one of the voluntary schemes introduced in the UK (2011) where local governments support food catering outlets to improve the nutritional quality of meal offerings [35].

The HCC [27,36] aims to encourage food businesses to reduce saturated fat, salt, and sugar in food sold on their premises and offer healthier choices and smaller portions. However, there is limited evidence on the effectiveness of these interventions, especially in terms of outcome evaluation [36]. Thus, this study aimed to determine the total fat content and fatty acid profiles of takeaway meals from ‘standard’ and HCC-approved food outlets.

## 2. Materials and Methods

### 2.1. Data Collection

We conducted a cross-sectional study across 8 London (UK) boroughs to represent different socioeconomic characteristics [37]. The HCC scheme is open to all types of food catering establishments, including restaurants, canteens, cafés, takeaways, bars, pubs, sandwich bars, caterers, and leisure centers in London. This study focused only on takeaways as they are one of the most popular out-of-home meal types consumed in the UK [38,39]. To identify takeaway outlets that hold the HCC award, the HCC database listing participating food businesses (available at https://healthiercateringcommitment.co.uk/find-businesses/ (accessed on 5 May 2019) was searched. The researchers also examined all London borough HCC websites and contacted local borough HCC contacts. Additionally, during the takeaway visit, an incognito researcher confirmed that a window sticker featuring the HCC logo and/or the HCC certificate was displayed to ensure that a takeaway business was participating in the HCC scheme. All meals were anonymously purchased from 27 (14 ‘standard’ and 13 ‘HCC-awarded’, including Chinese, fish and chips, kebab, and chicken shops) selected takeaway outlets. The HCC meal and corresponding standard meal were purchased at the same time and location to ensure consistency. The collection of meals took place between June 2019 and July 2019. All meals were served hot, were freshly prepared on the site, and were purchased once from each selected takeaway outlet. In total, 26 side dishes (chips and egg fried rice) and 74 meals (Chinese: sweet and sour chicken, black bean chicken, special fried rice, chow mein; fish and chips; kebab; burger; and fried chicken) were purchased. Chinese and fish and chips meals were selected based on their popularity amongst UK adult consumers and fried chicken and burgers were selected amongst adolescents [16,40,41].

### 2.2. Sample Analysis

All meals were kept in their packaging during transportation to the laboratory where they were weighed on the day of collection, homogenized, and stored at −80 °C until analysis. All visited takeaway outlets that served fish and fried chicken with chips were separated into their component parts of fish, chicken (bones removed), and chips before nutritional analysis. All sampled meals were analyzed for total fat (g) and fatty acid composition, including saturated fatty acids (g), monounsaturated fatty acids (g), polyunsaturated fatty acids (g), and trans fatty acids (g) by Premier Analytical Services (High Wycombe, UK) UKAS (United Kingdom Accreditation Service), an accredited food analysis laboratory. Total fat was determined by nuclear magnetic resonance after the samples were dried [42]. Triacyl glycerides were extracted by ethyl and petroleum ethers and then saponified to release the individual fatty acids. Fatty acids were transesterified to form fatty acid methyl esters in preparation for separation by GC analysis (polar capillary column) and quantification using a flame ionization detector [43].

### 2.3. Statistical Analysis

The data were analyzed using SPSS version 29.0 (SPSS Inc., Chicago, IL, USA), and *p* < 0.05 was considered statistically significant. All variables were checked for normality of distribution using histograms, Kolmogorov–Smirnov, and Shapiro–Wilk tests. Due to nonparametric distribution, all results are presented as medians with interquartile range (25th and 75th percentiles) per 100 g and portion. The takeaway meals within HCC and standard meal groups were divided into three meal categories according to their origin: 1. Chinese, 2. English, 3. Kebabs. Differences in fat and fatty acid levels between HCC and standard meals were tested with the use of the Mann–Whitney U-test. Differences in nutritional quality between meal categories and different types of meals within categories were analyzed using the Kruskal–Wallis and Dunn’s tests. The total fat, SFAs, PUFAs, MUFAs, and TFAs levels in takeaway meals were compared with the UK Dietary Reference Values (DRVs) for men and women aged 19–50 years [44,45,46]. In addition, meals were also classified according to the UK Food Standards Agency (FSA) front-of-pack labeling scheme, which is a voluntary color coded ‘Traffic Light’ system, with the color red indicating high, amber indicating medium, and green indicating low contents of total fat, SFAs, sugar, and salt [47].

## 3. Results

The median fat and fatty acid levels per 100 g and per portion in HCC-approved and standard takeaway meals are presented in Table 1. On average, the standard meals were characterized by significantly bigger portion sizes (580 vs. 511 g; *p* < 0.05) when compared to HCC meals, but no statistically significant differences in total fat, SFAs, MUFAs, PUFAs, and TFAs per 100 g and per portion between HCC and standard meals were observed. However, the nutritional profile varied considerably between the takeaway categories. Kebabs and English meals were significantly higher in total fat, SFAs, MUFAs, and TFAs per 100 g and per portion when compared to Chinese meals, with the same trend observed in standard and HCC meal groups. It is worth noting that there were no statistically significant differences in the nutritional profile per 100 g and per portion when comparing a HCC Chinese to standard Chinese meals and HCC English to standard English meals. However, interestingly, HCC kebabs were higher in total fat and SFAs per 100 g when compared to standard kebabs.

Furthermore, significant differences in the fat content and fatty acid composition between different types of meals but from the same meal category were observed (Table 1). For example, among standard English meals, a portion of a burger provided three times less total fat (23.2 vs. 76.0 g/per portion), 50% less SFAs (9.6 vs. 18.7 g/per portion), and four times less TFAs (0.8 vs. 3.4 g/per portion) than a portion of fried chicken with chips. Similar observations were made in the HCC English meal category. The comparison of different types of HCC meals with corresponding standard meals showed that the HCC beef burger was significantly lower in SFAs and TFAs and that special chow mein was lower in total fat and PUFAs content per 100 g when compared to standard burgers and standard special chow mein, but no statistically significant differences per portion were reported. Moreover, it was observed that the portion size of standard fish and chips, chicken and chips, and kebabs was approximately 200 g larger than the portion size of corresponding HCC meals.

We also noted that similar types of meals, cooked in different takeaway outlets, varied considerably in their fat and fatty acid content (Table 1). For example, the total fat content in 100 g of special chow mein prepared in standard takeaway establishments varied between 2.2 g and 3.9 g. Similarly, a portion of fish and chips served in different HCC-awarded outlets contained between 7.3 and 15.7 g of total fat and between 1.1 and 2.5 g of SFAs per 100 g.

**Figure 1:**
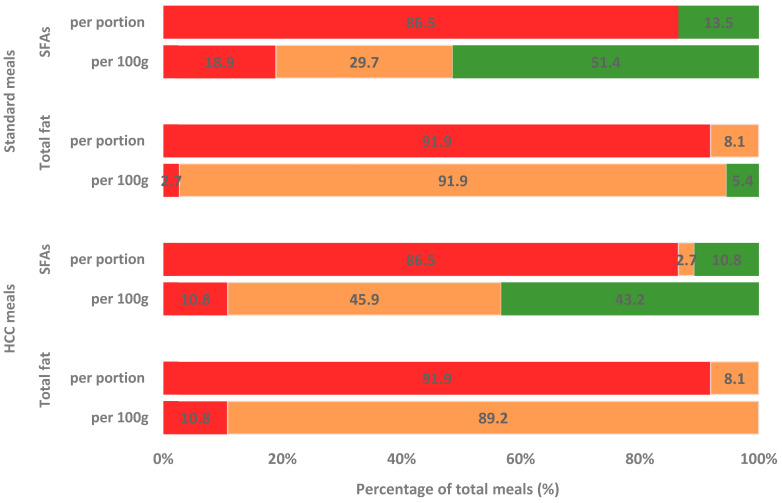
The comparison of the nutritional profile of takeaway meals to the Food Standard Agency front-of-pack traffic light labeling scheme. Red indicates high, amber indicates medium, and green indicates low content of total fat and SFAs [47].

The comparison of the nutritional profile of takeaway meals to the Food Standard Agency front-of-pack traffic light labeling system (Figure 1) revealed that only 5.4% of standard takeaway meals and none of the HCC-awarded meals were classified as ‘green’ (≤3 g/100 g) based on their total fat content per 100 g. When looking at the SFA level per 100 g, 43.2% and 51.4% of HCC and standard meals, respectively, met the criteria for a ‘green’ category (≤1.5 g/100 g). The situation was even worse when the total fat and SFA content per portion was analyzed, with 86.5% and 91.9% of takeaway meals assigned into a ‘red’ category (total fat > 21 g/portion; SFAs > 6 g/portion), respectively.

The fat and fatty acid content was also compared to the UK DRVs (Table 2). The standard meals provided, on average, 48% and 39% of the DRV for total fat and 35% and 28% of DRV for SFAs for women and men, respectively. Similarly, the HCC-awarded meals were high in total fat and SFAs that varied from 28 to 105% (average 49%) and from 22 to 84% (average 39%) of DRV for fat and from 27 to 171% (average 34%) and from 21 to 136% (average 26%) of DRV for SFAs for women and men, respectively. In addition, over 70% of all meals contained more than the recommended 30% of daily intake of fat from a single meal [48], and approximately a quarter of meals contained more than 30% of the recommended maximum intake of TFAs. It should be noted that the content of TFAs was very high in standard and HCC kebabs. The analyzed meals were high in PUFAs, with HCC meals providing 96 and 77% and standard meals providing 88 and 71% of the DRV for PUFAs for women and men, respectively.

## 4. Discussion

To the best of our knowledge, this is the first study to provide the outcome evaluation of this type of public health intervention. We observed that, overall, standard meals were characterized by significantly bigger portion sizes (580 vs. 511 g; *p* < 0.05) when compared to HCC meals. However, no statistically significant differences in total fat, SFAs, MUFAs, PUFAs, and TFAs per 100 g and per portion between HCC and standard meals were observed, except for donner kebabs. HCC donner kebabs were higher in total fat and SFAs per 100 g than standard kebabs. Similarly, a previous evaluation of the HCC scheme by Islington Council (London) in 2016 indicated that a reduction in saturated fats was reported in some HCC takeaway outlets, but revealed that more work is needed [49].

The present study supports previous research suggesting the poor nutritional quality of takeaway meals, which are often high in total fat and SFAs. Overall, 86.5% and 91.9% of HCC and standard takeaway meals were classified as ‘red’ according to the FSA traffic light system based on total fat and SFA content per portion, respectively. These results are consistent with our previous findings for meals offered in UK hospital canteens, where 34% and 40% of meat-based and vegetarian meals were classified as ’red’ for total fat and 67% and 80% were the same for SFAs, respectively. The highest content of total fat and SFAs was reported in chicken and chips and doner kebabs (Table 1). A single meal of doner kebab can provide over 80% of the recommended daily intake of total fat and over 130% of SFAs (Table 2). The total fat and SFAs contents per portion of kebab reported in this study were higher than previously observed by Davies et al. [50] in donner kebabs collected from takeaways in Liverpool, with 71.8 g/portion of total fat and 29.92 g/portion of SFAs, and in Scotland, with 29.7 g/portion of SFAs [51]. However, the amounts of total fat and SFAs per 100 g were similar. Interestingly, levels of total fat and SFAs found in doner kebabs sampled in Italy were 2–3 times lower than in the UK, with 8.5–8.7/g100 g and 2.1–2.3 g/100, respectively [52].

The out-of-home meals are a regular component of the Western diet, so they might significantly contribute to the overall intake of SFAs. Some communities might be more affected, especially people living in more deprived areas, where takeaway food outlet density is significantly higher [53]. The average intake of SFAs among UK adults is between 12.3 and 13.3% of total energy, which exceeds the recommended intake [44]. Changes to portion sizes present an opportunity to significantly influence the intake of total fat and SFAs among takeaway food consumers. Portion sizes were inconsistent not only between different takeaway meals but also across similar meals served in different takeaway outlets. This variation has influenced the nutritional profile of takeaway meals; for example, a portion of standard chicken and chips ranged from 450 to 603 g and provided between 68.5 and 93.7 g of total fat and 16.5 and 24.1 g of SFAs (Table 1). The provision of smaller portions (between 1/2 and 1/3 of the standard portion) for both children and adults is one of the HCC’s essential criteria that must be met by all businesses [36]. While some HCC takeaway outlets offered smaller portions than standard takeaways, there was no significant effect of portion size reduction on the total fat and SFA contents (Table 1), except for doner kebabs and chow mein. Although HCC doner kebabs and chow mein had higher total fat contents per 100 g than corresponding meals from standard takeaway outlets, the content per portion did not differ due to smaller portions of these meals in HCC businesses. Bagwell et al. [54] reported that some takeaway businesses might be encouraged to offer smaller portion sizes, but only in the areas where they were able to provide quality over quantity. The majority of the analyzed meals were excessive in portion size. However, it is unknown whether these meals are shared or consumed by one person. Therefore, further studies are required to identify the eating practices of takeaway meals consumers. Numerous studies have demonstrated that portion size has a powerful effect on the amount of food consumed and the same energy intake [55], and portion size management has been recognized as an effective strategy to address the increasing rates of obesity at the population level [56]. It was reported that portion size controlling is important to from one-third to one-half of consumers depending on their age and attitudes towards eating and health [57]. Among consumers surveyed in Northern Ireland, 48% and 46% indicated that they would like healthier food to be offered in takeaways and fast-food restaurants, respectively, and around two-thirds of consumers found it challenging to choose healthier meals when eating out. Interestingly, while consumers were likely to purchase food with reduced sugar, fat, and salt content, they were less likely to purchase food with reduced portion sizes [57]. Our results suggest that in addition to the changes in portion size, meal reformulation should be explored to improve the nutritional quality of takeaway foods. The substantial variability of the nutritional profile observed across similar types of takeaway meals provides an opportunity to improve by recipe reformulation and/or alternations in food preparation. In addition, the high variability in nutrient content may suggest a lack of standard procedures for meal preparation in takeaway outlets. Studies have shown that it is possible to modify takeaway meal recipes and introduce alternation in food preparation to enhance their nutritional quality without decreasing consumer acceptability [58]. Combet et al. [59] reformulated a Margherita pizza, and the total fat content was reduced to 15.7 g and the SFA content to 6.0 g per pizza. Importantly, 77% of adults and 81% of children rated it ’as good as’ or ’better than’ their usual choice [59]. Furthermore, Jaworowska et al. [60] reported that a 13–69% reduction in the fat content of chow mein meals was possible to achieve without any significant impact on their overall acceptability.

Our results surprisingly indicate a significant reduction in SFA content in some deep-fried meals compared to the results of previous studies. For example, we observed the average SFA content per portion of standard fish and chips was 9.2 g (1.3 g/100 g) and 7.1 g (1.2 g/100 g) per portion of HCC fish and chips. In contrast, the previous study conducted across takeaways in Liverpool [50] reported 41.99 g SFAs/portion, and similar levels were found in fish and chips meals collected from takeaway outlets in Scotland (27 g SFAs/portion) [51] and Sandwell (34 g SFAs/portion) [61]. The lower levels of SFAs were also found in chips (HCC: 1.1 g/100 g and 5.2 g/portion; standard: 1.5 g/100 and 4.6 g/portion) compared to those previously reported in Scotland (around 5.0 g/100 and 14 g/portion of chips) [51] and Sandwell (19.8 g/portion of chips) [61]. On the other hand, our results are similar to the SFA levels observed in chips from fast-food chains in the US [62]. As the total fat content in the analyzed deep-fried meals was comparable to the levels reported in previous studies, this suggests that the observed reduction in SFA content may be due to a change in the type of fat used for deep-fat frying. The reduction in saturated fat intake is an important public health strategy to improve cardiovascular health. It has been reported that lower SFA intake could reduce combined cardiovascular events by 17% [63]. In addition, reduced SFA intake had favorable effects on blood pressure and blood lipids in children [64]. Furthermore, a high intake of SFAs was correlated with an increased cancer risk, including breast, prostate, and colorectal cancers [65].

It is worth noting that some takeaway meals were characterized by high levels of MUFAs and PUFAs. Similar findings have been reported in takeaway food purchased in Bosnia and Herzegovina, with the average content of MUFAs being 3 g and PUFAs being 1.9 g per 100 g of food [66]. However, there is very limited data on the content of these fatty acids in takeaway meals, and the proportion between n-6 and n-3 PUFAs is unknown. Our results suggest that high levels of MUFAs and PUFAs in takeaway meals may be attributed to the type of fat used for frying. Thus, the high n-6/n-3 ratio in these meals may be expected. Vegetable oils rich in n-6 PUFA, such as rapeseed oil, soybean oil, sunflower oil, and corn oil, are commonly used for cooking deep-fried foods [67]. Evidence from randomized controlled trials suggests that replacing SFAs with mostly n-6 PUFAs is unlikely to reduce coronary heart disease events [68].

On a positive note, the majority of takeaway meals analyzed in the present study are unlikely to significantly contribute to the overall TFA intake, with their average TFA content being between 0 and 0.9 g/portion. However, HCC and standard doner kebabs contained more than the recommended maximum daily intake of TFAs (no more than 2% of total daily energy), with a median average of 5.5 and 6.1 g/portion of TFAs, respectively. Similarly to the present study, the FSA UK reported, in 2014, that the levels of TFAs in the deep-fried meals purchased from takeaway outlets in the most deprived areas of Scotland were low (average of 1.5 g TFAs per portion), except doner kebabs, which contained 0.89 g/100 g and 3.70 g/portion of TFAs [51]. Also, Caraher et al. [69] found that, on average, takeaway meals collected in London (Tawer Hamlet) contained less than 2% of energy from TFAs, but the content of TFAs in doner kebabs was higher, with an average of 0.84 g/100 g, the equivalent of 2.9% of total meal energy. However, the amount of TFAs in doner kebabs reported in our previous study was lower, at levels of 0.43 g/100 g and 1.99 g/portion. It is worth noting that all takeaway meals tested in the previous studies, except boiled rice and chicken curry, contained some amount of TFAs [50,69], whereas, in the present study, we observed TFA occurrence only in deep-fried food, hamburgers, and donner kebabs. There should be little naturally occurring TFAs in the analyzed takeaway meals, apart from doner kebabs and hamburgers, which consist of ruminant meat containing natural TFAs (around 3–9% of total fatty acids and mostly vaccenic acid; 50–80% of total trans fat) synthesized via the bacterial metabolism of unsaturated fatty acids in ruminant animals [70]. This indicates that the level of TFAs in deep-fried food depends on the type of fat used and/or cooking/frying practices. Whilst it is not fully clear why some of the analyzed deep-fried foods contained TFAs, it could be linked to the use of beef dripping; it was previously reported that the ‘fish and chip’ shops mostly used animal-origin fat for frying [51]. In addition, TFAs are formed in vegetable oils when heated to very high temperatures, during prolonged heating, or through the repeated use of vegetable oils that are reheated several times [71,72]. Roe et al. [73] reported that industry reformulations have significantly reduced the total TFA content of processed foods in the UK and that the content of elaidic acid from hydrogenated oils was low, with the exception of deep-fried takeaway food, including fish in batter, chips, and fried chicken pieces. Similarly, Wagner et al. [74] observed, in Austria, a significant reduction in the average TFA content in French fries (collected from fast-food outlets), from 3.3 g/110 g to 0.3 g/100 g between 1996 and 2006. These levels are comparable to our observation of 0.2 g/100 g of TFAs in standard chips, considerably lower than the TFA content in takeaway chips (1.14 g/portion) reported by the FSA in 2014 [51]. A similar trend was observed in McDonalds’ chicken nuggets and French fries in London, UK, where the TFA content was reduced from 7 g to less than 1 g between 2005 and 2009 [75], as well as in French fries available from five Spanish fast-food chains [76].

This study showed no significant differences in the nutritional quality of takeaway meals served in standard takeaways compared to those awarded the HCC certificate. It is unclear why the nutritional quality has not been improved. A previous evaluation of the HCC scheme revealed that the majority of catering businesses were not interested in providing healthier options, such as fresh fruits and wholegrain products, especially those located in less affluent areas, as the healthier alternatives were not popular with consumers [36]. However, some businesses also found that offering baked potatoes, poached fish, and salads might attract new customers who were on a diet [54]. Bagwell et al. [32] reported that the HCC scheme resulted in the provision of healthier out-of-home meals for more affluent consumers only, with healthier meal alternatives not being accepted in the more deprived communities, especially if they resulted in increased prices. Even simple changes might be difficult to implement for businesses operating in less affluent communities, as the additional costs of healthier meals cannot be passed on to customers [32]. Similarly, Goffe et al. [77] found that fish and chip shops serving higher priced meals, which may reflect a more affluent customer base, were more likely to use reduced-holed shakers to lower the amount of salt added to meals [77]. Moran et al. [59] observed no substantial changes in calories, sodium, or saturated fatty acids in children’s meals served in chain restaurants participating in the Kids LiveWell initiative and non-participating restaurants.

The modification of the product surface is one of the most effective methods for reducing fat uptake in deep-fried food products [78,79]. A larger surface of chips results in proportionally higher absorption of fat, with thick (>12 mm) and straight cut chips shown to have a lower fat content [80,81]. Frying at the optimal temperature, maintaining the quality of the frying fat, and post frying de-oiling, for example by vigorously shaking the basket and hanging it over the deep fryer to drain excess surface fat, are also important factors in controlling the fat content of deep-fried food [78,79,80]. Some of the HCC recommended changes include the proper maintenance and heating of cooking oil to optimum temperatures, offering thick-cut chips, and the adequate draining of excess fat from food after cooking [36]. However, it is not known whether the takeaway business followed the HCC-recommended deep-fat frying practices. The local government staff involved in the HCC implementation recognized that maintaining the HCC standards may be a challenge, and without ongoing pressure, businesses might return to their old modes of operation [82]. Moreover, it is important that all deep-fat frying operators are adequately trained in deep-fat frying techniques; however, Morley-John et al. [80] reported that only 8% of independent food outlet staff in Australia were trained in deep-fat frying practices. Data could not be located for UK independent food outlets.

This study has several limitations. While it evaluated the fat content and fatty acids profile of takeaway meals, a more comprehensive analysis, including energy, carbohydrates (including simple sugars), fiber, and sodium levels would provide a more detailed nutritional profile of these meals. In addition, only one sample of each meal was analyzed, and only a limited variety of takeaway meal types was included; therefore, our results might not precisely determine the nutritional quality of takeaway meals. An analysis of multiple samples of the same meal from the same takeaway business and the inclusion of a wider variety of meals could provide more insight into their nutritional profile. Furthermore, an analysis of the nutritional quality of meals before and after the implementation of interventions as well as a collection of additional information on cooking practices and the ingredients used would be recommended.

Our results provide evidence to inform the development, implementation, and evaluation of interventions to increase the provision of healthier out-of-home meals. The observed significant variability of fat and fatty acids content across similar takeaway meals suggests the possibility of improving their nutritional quality by recipe reformulation and/or alternations in food preparation and should be further explored. In addition, the provision of nutrition labels might help consumers choose meals with a more favorable nutritional profile and improve dietary intake when eating out of the home. However, in the UK, small, independent takeaway establishments are not required to provide nutritional labeling; thus, consumers may find it extremely difficult to recognize the nutritional composition of these meals. Therefore, it is important to encourage takeaway establishments to display nutrition information on their menus to help consumers make healthier food choices. Young et al. [83] suggested the potential benefits of menu labeling on the nutritional composition of menu items. It was observed that meals from restaurants required to follow menu labeling regulations were smaller in portion size and lower in energy and nutrients of public health concern than those from unregulated restaurants. Additionally, reducing portion sizes or suggesting that some meals are suitable for sharing could improve overall dietary intake.

Furthermore, our results, similarly to the findings of previous studies, suggest that more intrusive interventions that restrict, eliminate, or guide choice through incentives/disincentives might be more effective than policies that provide information or enable choice. Our findings indicate that voluntary initiatives might not be enough to significantly improve the nutritional profile of this type of food, and some additional government regulations seem to be needed to encourage wider participation including the supply chain. The government and local authorities have a crucial role in enabling food outlets to promote healthier meals and in enforcing that nutrition claims comply with the regulations, ensuring consumers are not misled. Coordinated efforts between food outlets, suppliers, researchers, and public health organizations might be the most effective way to improve the nutritional quality of takeaway meals, ensuring that costs, taste, and acceptance are not compromised. In addition, this study suggests that constant monitoring and evaluation are needed to maintain the standards of implemented interventions.

## 5. Conclusions

The results of this study indicate that, despite a number of businesses participating in the healthier out-of-home meal initiatives, there has not been a significant improvement in the nutritional quality of the meals they offer. Therefore, further research to develop effective approaches to support independent takeaway outlets in offering meals with improved nutritional quality is warranted.

## Figures and Tables

**Table 1:** The fat content and fatty acid composition of takeaway meals.

Meals (*n*)	Total Fat (g/100 g)	SFAs (g/100 g)	MUFAs (g/100 g)	PUFAs (g/100 g)	TFAs (g/100 g)	Portion Size (g)	Total Fat (g/Portion)	SFAs (g/Portion)	MUFAs (g/Portion)	PUFAs (g/Portion)	TFAs (g/Portion)
Standard meals (*n* = 37)	8.6 (5.4–13.0)	1.4 (1.0–4.2)	3.2 (1.8–5.5)	2.1 (1.5–2.7)	0.04 (0.0–0.4)	580 (520–770) ^X^	52.0 (29.8–75.8)	10.1 (6.6–16.7)	17.9 (10.1–29.7)	13.3 (6.3–21.9)	0.4 (0.0–1.1)
Chinese (*n* = 16)	5.2 (3.9–6.0) ^2,3^	1.0 (0.7–1.1) ^2,3^	1.7 (1.3–2.0) ^2,3^	2.1 (1.7–2.5)	0 (0–0) ^2,3^	758 (562–998) ^2,3^	37.9 (26.4–53.6) ^3^	6.6 (5.8–10.2) ^2,3^	13.1 (9.1–18.4) ^3^	16.1 (10.8–24.8)	0 (0–0) ^2,3^
Beef in black bean sauce with egg fried rice (*n* = 4)	5.0 (4.1–5.7)	0.9 (0.7–1.2)	1.7 (1.2–2.0)	2.0 (1.9–2.4)	0 (0–0)	998 (909–1150) ^h^	48.4 (40.6–61.2)	8.9 (6.5–12.7)	17.6 (13.4–20.7) ^h^	20.1 (17.4–25.7)	0 (0–0)
Sweet and sour chicken with egg fried rice (*n* = 4)	6.2 (5.5–7.8) ^h^	1.1 (0.9–1.3)	1.9 (1.7–2.4)	3.0 (2.3–3.7) ^h^	0.02 (0.00–0.04)	990 (890–1073)	60.3 (51.9–77.2) ^h^	10.5 (8.1–13.2) ^h^	18.8 (17.3–23.1) ^h^	29.5 (23.6–36.3) ^h^	0.2 (0.0–0.5)
Special fried rice (*n* = 4)	5.5 (4.9–6.7)	1.1 (1.1–1.6)	1.7 (1.5–2.8)	2.2 (1.9–2.5)	0 (0–0)	579 (561–624)	32.2 (30.2–36.5)	6.4 (6.3–6.9)	10.1 (9.8–14.0)	12.5 (12.0–14.1)	0.0 (0.0–0.3)
Special chow mein (*n* = 4)	3.0 (2.2–3.9) *	0.7 (0.9–1.7)	0.9 (0.6–1.4)	1.2 (0.9–1.5) *	0 (0–0)	551 (525–594)	17.3 (12.2–22.4)	3.6 (2.4–4.9)	5.3 (3.5–7.7)	6.4 (5.1–8.6)	0 (0–0)
English (*n* = 17)	12.3 (9.8–14.9)	3.6 (3.1–4.2)	4.9 (3.9–6.0)	2.3 (1.5–4.1)	0.3 (0.1–0.5) ^3^	540 (232–674)	65.8 (29.5–76.0)	11.2 (9.0–18.7)	18.2 (11.2–35.7)	15.1 (4.3–21.9)	0.8 (0.4–2.0
Beef burger (*n* = 6)	11.5 (9.9–14.0)	4.5 (2.8–3.7) ^k^	4.7 (3.8–6.6)	1.2 (0.8–2.0) ^j,k^	0.3 (0.2–0.4) *	207 (183–234) ^k^	23.2 (21.3–29.5) ^j,k^	9.6 (8.2–11.2)	9.0 (8.2–14.6) ^j,k^	2.5 (1.6–4.3) ^j,k^	0.8 (0.5–0.8)
Chicken and chips (*n* = 7)	14.9 (12.6–16.2)	3.5 (2.9–4.2)	5.5 (5.1–5.3)	3.2 (2.3–4.1)	0.5 (0.1–1.4)	566 (450–603) *	76.0 (68.5–93.7)	18.7 (16.5–24.1)	35.7 (26.3–36.6)	18.4 (14.4–23.1)	3.4 (0.3–7.1)
Fish and chips (*n* = 4)	9.2 (7.9–11.3)	1.3 (1.1–2.5)	3.4 (2.3–4.5)	3.4 (2.3–3.7)	0.1 (0.0–0.2)	767 (722–852) *	70.8 (60.0–89.9)	10.4 (7.0–23.1)	25.1 (17.8–35.9)	27.8 (18.5–38.4)	1.0 (0.1–2.0)
Kebab (Doner kebab) (*n* = 4)	15.5 (14.4–17.5) *	6.8 (7.7–13.1)	5.6 (5.3–6.5)	1.3 (0.7–1.8)	1.1 (0.9–1.2)	568 (479–668) *	92.0 (76.8–104.2)	40.4 (35.1–44.0)	34.3 (28.5–38.1)	6.5 (4.9–10.0)	6.1 (4.8–7.5)
Sides (*n* = 13)	8.6 (6.4–10.1)	1.2 (0.8–1.6)	2.5 (1.9–3.4)	2.9 (1.9–4.6)	0.0 (0.0–0.2)	480 (290–524)	26.2 (22.8–40.1)	4.6 (3.4–7.5)	9.2 (7.6–11.9)	11.4 (10.7–15.4)	0 (0–0.6)
Chips (*n* = 9)	9.3 (8.5–11.2) ^l^	1.5 (0.9–2.6) ^l^	3.3 (2.4–4.6) ^l^	4.4 (2.1–5.0)	0.2 (0.0–0.2) ^l^	400 (256–495) ^l^	32.5 (22.8–42.5)	4.6 (3.3–7.7)	9.2 (7.6–14.3)	11.7 (11.2–15.4)	0.5 (0.0–0.9) ^l^
Egg fried rice (*n* = 4)	4.5 (4.0–6.0)	0.8 (0.7–0.9)	1.6 (1.1–2.1)	1.9 (1.7–2.6)	0 (0–0)	558 (499–617)	24.2 (22.0–32.5)	4.2 (3.6–6.1)	9.3 (6.7–11.3)	10.6 (8.9–14.7)	0 (0–0)
HCC meals (*n* = 37)	8.0 (6.1–12.7)	1.8 (1.1–3.2)	2.6 (1.9–5.3)	2.3 (1.9–3.3)	0.0 (0.0–0.2)	511 (384–614) ^X^	43.6 (31.2–57.0)	8.4 (6.3–13.8)	13.7 (10.3–23.8)	14.4 (7.4–19.4)	0.0 (0.0–0.5)
Chinese (*n* = 16)	6.1 (5.0–6.8) ^2,3^	1.0 (0.9–1.4) ^2,3^	1.9 (1.5–2.4) ^2,3^	2.3 (2.2–3.1)	0 (0–0.1) ^2,3^	621 (532–804) ^2,3^	37.9 (32.0–46.7) ^3^	7.7 (5.8–8.9) ^3^	12.2 (9.3–15.9) ^3^	16.4 (13.6–20.1) ^3^	0 (0–0.4) ^2,3^
Beef in black bean sauce with egg fried rice (*n* = 4)	4.7 (4.3–5.1)	0.8 (0.7–0.9)	1.4 (1.2–1.9)	2.1 (2.0–2.3)	0 (0–0)	820 (671–1016) ^h^	38.1 (32.0–46.6)	7.0 (5.8–8.4)	11.1 (9.3–15.4)	17.7 (15.2–20.1)	0 (0–0)
Sweet and sour chicken with egg fried rice (*n* = 4)	6.1 (6.1–7.6)	0.3 (0.9–1.3)	2.0 (1.6–2.6)	3.0 (2.5–3.6)	0.04 (0.01–0.05)	804 (636–1027)	56.1 (46.4–60.7)	8.9 (7.0–10.3)	16.9 (13.6–20.4)	24.1 (20.0–29.6) ^f,h^	0.4 (0.2–0.5)
Special fried rice (*n* = 4)	6.1 (5.7–7.1)	1.4 (1.1–1.6)	2.2 (1.9–2.4)	2.3 (2.3–2.9)	0 (0–0)	593(402–620)	35.0 (29.1–37.6)	7.3 (6.2–8.3)	11.6 (9.2–13.8)	13.7 (12.1–14.1)	0 (0–0)
Special chow mein (*n* = 4)	6.5 (4.6–7.9)	1.4 (0.9–1.7)	2.0 (1.4–2.5)	2.7 (2.0–3.2)	0 (0.0–0.1)	514 (390–539)	30.7 (20.4–41.6)	6.2 (4.1–9.1)	9.8 (6.4–13.1)	13.3 (8.9–17.0)	0 (0–0.3)
English (*n* = 17)	11.6 (8.6–13.0)	3.0 (2.7–3.7)	5.1 (3.3–5.6)	3.2 (1.5–3.8)	0.1 (0.0–0.2) ^3^	438 (240–514)	50.7 (25.4–57.0) ^3^	8.9 (7.8–13.9) ^3^	14.5 (10.2–23.8)	13.3 (6.0–20.5)	0.4 (0.0–0.9)
Beef burger (*n* = 6)	9.9 (7.8–11.5)	3.4 (2.8–3.7)	3.9 (3.3–5.5)	1.2 (1.0–2.7) ^k^	0.1 (0.07–0.2)	230 (215–245) ^j,k^	22.5 (20.8–25.4) ^j^	8.0 (6.3–8.4) ^j^	9.1 (8.6–11.4) ^j^	2.9 (2.5–6.0) ^j,k^	0.3 (0.2–0.5) *
Chicken and chips (*n* = 7)	13.0 (11.9–14.1)	3.1 (2.9–4.2)	5.3 (5.1–6.1)	3.3 (2.0–3.8)	0.2 (0.1–1.1)	364 (404–514)	57.0 (52.9–66.5)	13.9 (13.5–17.8)	23.8 (22.1–29.4)	15.4 (10.6–17.2)	0.9 (0.5–3.0)
Fish and chips (*n* = 4)	8.0 (7.3–15.7)	1.2 (1.1–2.5)	2.2 (1.8–7.8)	4.0 (3.8–4.4)	0.0 (0–0.6)	559 (477–659)	46.2 (40.2–75.3)	7.1 (5.8–12.2)	12.2 (10.8–32.9)	21.5 (20.5–25.0)	0 (0–2.4)
Kebab (Doner kebab) (*n* = 4)	20.6 (17.6–27.8)	9.5 (7.7–13.1)	7.2 (6.5–10.1)	1.6 (1.5–1.8)	1.3 (0.8–1.8)	387 (314–437) ^k^	85.1 (75.9–86.5)	39.4 (33.7–40.7)	6.0 (5.0–7.4)	6.9 (5.4–7.3)	5.5 (4.1–5.6)
Sides (*n* = 13)	7.3 (5.0–12.4)	1.0 (0.8–1.8)	1.9 (1.7–4.4)	3.1 (2.0–3.9)	0 (0–0)	440 (340–536)	35.6 (21.9–43.6)	4.6 (3.1–5.5)	10.2 (5.9–13.8)	11.6 (10.4–17.6)	0 (0–0)
Chips (*n* = 9)	9.9 (6.8–13.7) ^l^	1.1 (0.9–2.6) ^l^	3.7 (1.8–5.4) ^l^	3.4 (2.9–5.7) ^l^	0 (0–0.6)	440 (268–563)	38.8 (34.7–50.0) ^l^	5.2 (4.3–8.2) ^l^	10.9 (9.4–16.4) ^l^	16.6 (11.6–19.8) ^l^	0 (0–0.9)
Egg fried rice (*n* = 4)	4.3 (4.1–4.8)	0.8 (0.7–0.9)	1.2 (1.1–1.7)	2.0 (2.0–2–2)	0 (0–0)	439 (341–535)	18.4 (14.9–24.3)	3.2 (2.6–4.3)	5.2 (4.3–8.)	9.3 (7.4–10.7)	0 (0–0)

^X^ significant difference between HCC and standard meals (Mann–Whitney U test, *p* < 0.05); * significant difference between HCC and corresponding standard meals (Mann–Whitney U test, *p* < 0.05); ^2^ English, ^3^ kebab; ^f^ sweet and sour chicken with rice, ^h^ special chow mein, ^j^ fried chicken and chips, ^k^ fish and chips, ^l^ egg fried rice; *n*—number; SFAs—saturated fatty acids; MUFAs—monounsaturated fatty acids; PUFAs—polyunsaturated fatty acids; TFAs—trans fatty acids.

**Table 2:** Total fat and fatty acid levels of takeaway meals compared to the UK daily reference values for healthy adults.

Meals (*n*)	Total Fat (%DRV Women)	SFAs (%DRV Women)	MUFAs (%DRV Women)	PUFAs (%DRV Women)	TFAs (%DRV Women)	Total Fat (%DRV Men)	SFAs (%DRV Men)	MUFAs (%DRV Men)	PUFAs (%DRV Men)	TFAs (%DRV Men)
Standard meals (*n* = 37)	64 (37–94)	44 (29–73)	64 (36–107)	88 (42–146)	9 (0–43)	51 (29–75)	35 (23–58)	51 (29–85)	71 (34–117)	8 (0–35)
Chinese (*n* = 16)	47 (33–66)	29 (25–44) ^2^	47 (33–68)	116 (78–179)	0 (0–0) ^2^	37 (26–53)	23 (20–35) ^2^	38 (26–54)	93 (62–143)	0 (0–0) ^2^
Beef in black bean sauce with egg fried rice (*n* = 4)	59 (50–75)	38 (28–55)	63 (48–74)	145 (125–185)	0 (0–0)	48 (40–61)	31 (22–44)	51 (38–59)	116 (100–148)	0 (0–0)
Sweet and sour chicken with egg fried rice (*n* = 4)	74 (64–95)	45 (35–58)	68 (62–83)	213 (170–261)	5 (0–10)	60 (51–76)	36 (28–46)	54 (50–66)	170 (136–209)	3.8 (0–8)
Special fried rice (*n* = 4)	40 (37–45)	30 (27–30)	36 (35–51)	90 (86–102)	0 (0–6.0)	32 (30–36)	22 (22–24)	29 (28–40)	72 (69–81)	0 (0–5)
Special chow mein (*n* = 4)	21(15–28) ^f^	15 (11–21) ^f^	19 (13–28) ^f^	46 (37–62) ^f^*	0 (0–0)	17 (12–22) ^f^	12 (8–17) ^f^	15 (10–22) ^f^	37 (29–50) ^f^	0 (0–0)
English (*n* = 17)	81 (36–94)	48 (39–81) ^3^	66 (40–128)	108 (31–158)	18 (8–44)	65 (29–75)	38 (31–64) ^3^	52 (32–102)	87 (24–126)	14 (6–35)
Beef burger	29 (26–36) ^j,k^	42 (35–48)	32 (30–53) ^j^	18 (12–31) ^j,k^	17 (10–18)	22 (21–29) ^j,k^	33 (28–38)	26 (24–42) ^j^	14 (9–25) ^j,k^	13 (8–15)
Chicken and chips	93 (84–116)	81 (71–105)	128 (95–132)	132 (103–166)	73 (6–153)	75 (68–93)	64 (57–83)	102 (76–105)	106 (83–133)	58 (5–122)
Fish and chips	87 (74–111)	45 (30–100)	90 (64–129)	200 (133–277)	22 (0–44)	70 (59–89)	36 (24–80)	72 (51–103)	160 (107–222)	17 (0–35)
Kebab (doner kebab) (*n* = 4)	114 (95–129) ^1,3^	175 (152–181) ^1,3^	123 (103–137) ^1,3^	45 (35–72)	132.2 (0.23–0.36) ^1,3^	91 (76–103) ^1,3^	139 (121–152) ^1,3^	98 (82–109) ^1,3^	38 (28–57)	106 (83–129) ^1,3^
Sides	32 (28–49)	20 (15–33)	33 (27–43)	82 (77–111)	0 (0–12)	26 (23–40)	16 (12–26)	26 (22–34)	66 (62–89)	0 (0–10)
Chips	40 (28–52)	20 (14–33)	33 (27–51)	84 (81–111)	10 (0–20)	32 (22–42)	16 (11–26)	26 (22–41)	68 (65–89)	8 (0–16)
Egg fried rice	30 (27–40)	18 (17–27)	33 (24–41)	76 (64–106)	0 (0–0)	24 (22–32)	14 (12–21)	27 (19–33)	61 (51–85)	0 (0–0)
HCC meals (*n* = 37)	54 (38–70)	36 (27–60)	49 (37–85)	96 (49–130)	5 (0–12)	43 (31–56)	29 (22–48)	39 (30–68)	77 (39–103)	4 (0–9)
Chinese (*n* = 16)	47 (39–58)	33 (25–39)	44 (34–57)	118 (98–145)	0 (0–4)	37 (32–46)	27 (20–31)	35 (27–46)	95 (78–115)	0 (0–3)
Beef in black bean sauce with egg fried rice (*n* = 4)	47 (39–57)	30 (25–36)	40 (34–55)	127 (109–145)	0 (0–0)	38 (32–46)	24 (20–29)	32 (27–44)	102 (89–116)	0 (0–0)
Sweet and sour chicken with egg fried rice (*n* = 4)	69 (57–75)	38 (30–45)	61 (49–73)	173 (144–213)	8 (4–10)	55 (46–60)	31 (24–36)	49 (39–59)	139 (115–171)	6 (3–8)
Special fried rice (*n* = 4)	43 (36–46)	32 (27–36)	42 (33–50)	98 (87–101) ^f^	0 (0–0)	35 (29–37)	25 (21–29)	33 (26–40)	79 (69–81) ^f^	0 (0–0)
Special chow mein (*n* = 4)	38 (25–51)	27 (18–39)	35 (23–47)	96 (64–123) *	0 (0–5)	30 (20–41)	21 (14–31)	28 (18–37)	77 (51–98)	0 (0–5)
English	63 (31–70)	39 (34–60) ^3^	52 (37–86)	96 (43–148)	9 (0–20)	50 (25–56)	31 (27–48)	42 (29–68) ^3^	77 (35–118)	7 (0–16)
Beef burger	28 (26–31) ^j^	35 (27–36) ^j^	33 (31–41) ^j^	21 (18–43) ^k^	7 (5–10)	22 (21–25) ^j^	28 (22–29) ^j^	26 (25–33) ^j^	17 (14–35) ^k^	6 (4–8)
Chicken and chips	70 (65–82)	60 (59–77)	86 (80–106)	111 (76–124)	20 (10–65)	56 (52–66)	48 (46–61)	68 (63–85)	89 (61–99)	16 (8–52)
Fish and chips	57 (50–93)	31 (25–53)	44 (39–118)	155 (148–180)	0 (0–51)	46 (40–75)	24 (20–42)	35 (31–94)	124 (118–144)	0 (0–41)
Kebab (doner kebab)	105 (94–107) ^1,3^	171 (146–177) ^1,3^	108 (99–111) ^1,3^	50 (39–53) ^1^	118 (88–122) ^1,3^	84 (75–86) ^1,3^	136 (116–140)	86 (79–89) ^1,3^	40 (31–42) ^1^	95 (70–97) ^1,3^
Sides	43 (27–54)	20 (13–24)	37 (21–50)	83 (75–127)	0 (0–0)	34 (22–43)	16 (11–19)	29 (17–40)	67 (60–101)	0 (0–0)
Chips	48 (42–62) ^l^	23 (18–37)	39 (34–59) ^l^	119 (82–142) ^l^	0 (0–19)	38 (34–49) ^l^	18 (15–28)	31 (27–47) ^l^	96 (67–114) ^l^	0 (0–15)
Egg fried rice	23 (18–30)	14 (11–19)	19 (15–29)	67 (53–77)	0 (0–0)	18 (15–24)	11 (9–15)	15 (12–23)	53 (42–62)	0 (0–0)

* significant difference between HCC and corresponding standard meals (Mann–Whitney U test, *p* < 0.05), ^1^ Chinese, ^2^ English, ^3^ kebab, ^f^ sweet and sour chicken with rice, ^j^ fried chicken and chips, ^k^ fish and chips, ^l^ egg fried rice; *n*—number; SFAs—saturated fatty acids; MUFAs—monounsaturated fatty acids; PUFAs—polyunsaturated fatty acids; TFAs—trans fatty acids.

## Data Availability

Data is available on request.
